# Book review of “Joining and assembly of medical materials and devices” edited by Y. (Norman) Zhou and Mark D. Breyen

**DOI:** 10.1186/1475-925X-12-89

**Published:** 2013-09-08

**Authors:** Solomon P Samuel, Nathan C Tiedeken

**Affiliations:** 1 Department of Orthopaedic Surgery, Einstein Medical Center, Philadelphia, PA 19141, USA

**Keywords:** Biomaterials, Medical device assembly, Medical device joining, Welding, Bonding, Tissue adhesives

## Abstract

This article is a review of the book “Joining and assembly of medical materials and devices” edited by Y. (Norman) Zhou and Mark D. Breyen. This book (hardcover) was published by Woodhead Publishing, Cambridge, UK in 2013. The contents of the book and its relevance to medical device design and education are discussed in this invited review.

## Book details

Joining and assembly of medical materials and devices. Woodhead Publishing. Oxford, England. Y. (Norman) Zhou and Mark D. Breyen. 2013; 580 pages. List price: USD 290.00. ISBN-10 1845695771 (Hardback).

“Joining and assembly of medical materials and devices” was released in 2013 and edited by Y. (Norman) Zhou and Mark D. Breyen. This is the first edition and is dedicated to the understanding and assembly of implantable medical devices. Woodhead Publishing released 65 texts in the subject of biomaterials, and “Joining and assembly of medical materials and devices” is the 54^th^ text published in this biomaterial series. The stated objective of this book is to serve as a review of biomaterial concepts and a technical guide for engineers and researchers working in the biomedical industry. The chapters were written by authors who work in both industry and academia. The editors are well qualified and hold many publications and patents in their respective fields. Y. (Norman) Zhou also edited a similar but more general book titled “Microjoining and nanojoining” in 2008 (ISBN-10: 1420070835) [1].

The global implant and tissue adhesive market is currently well over $100 billion and continues to develop at a rapid pace. With this increased demand, there is a need to design medical devices that can be assembled in a cost effective way and function in one of the most corrosive environments. Constructing a reliable medical device requires a deep knowledge of biomaterials, welding techniques, techniques to maintain a sealed environment to protect electronic components and wires, effect of sterilization procedures on various components, effect of fatigue on joints or interfaces, testing/validation of various joints and corrosion prevention after implantation. This text is the first of its kind to confront the intricate relationship of biomaterials, assembly concepts and biology in a comprehensive textbook.

This 18 chapter book is divided into four parts namely, (a) fundamentals of joining and assembly, (b) joining and assembly of medical metals, (c) joining and assembly of medical plastics, (d) joining and assembly of metal-ceramic implants and review of various tissue adhesives. Each chapter is divided into several sections and begins with an abstract followed by a brief introduction to the specific topic. Illustrations, schematic diagrams, compatibility tables between different biomaterials, photographs, and equations are placed throughout each chapter to further aid in the understanding of each topic. All photographs are black and white with the exception of a four page color photograph section. Several of the chapters also have a brief section on future trends. This manuscript provides hundreds of references and a comprehensive review of practical biomaterial concepts and assembly techniques.

In the following paragraphs we highlight several chapters in “Joining and assembly of medical materials and devices.” Part one is composed of four chapters and serves as the main introduction for the text. All these four chapters describe the uniqueness of the biologic environment and explain how the requirements for joining implantable devices are very different from non implantable devices. For example, one can weld two biocompatible materials and end up with a non-biocompatible joint during the welding or sterilization process. These chapters provide a general overview of various joining methods utilized in the construction of medical devices including, but not limited to, laser welding, ultrasonic welding and adhesive bonding. A practical application of ultrasonic welding is its use in creating knotless sutures. A chapter is dedicated for microwelding and provides a brief summary of the unique joining challenges faced during assembly of various medical such as catheters, cochlear implants, pacemakers, neurosimulators, radioactive seed implants, etc. Sterilization is unique to the biomedical industry and the process itself can compromise the biocompatibility of the finished medical device. The effects that different sterilization has on various materials are reviewed in the fourth chapter. Detailed tables in that chapter provide a comprehensive review of many biomaterials and their compatibility to the described sterilization techniques.

Part two includes five chapters focusing on microwelding and other metal joining techniques. Three chapters were dedicated to joining Nitinol shape memory alloys, joining of dissimilar metals in medical devices (e.g. joining platinum alloy and stainless steel) and laser hermetic sealing technique. The other two chapters provide a guide for evaluating corrosion in welds and validating hermetic seals in metallic implants. Part three transitions to the welding techniques used in medical plastics. These chapters provide an overview of several plastic welding techniques (e.g. ultrasonic welding, radio frequency/dielectric welding and transmission laser welding) and testing. Chapter 14 reviews bonding strategies and adhesives for joining complex components in medical devices. This chapter seems out of place in part three and should have been placed with part 4. Part four contains four chapters that describe joining and assembling ceramic implants and various tissue adhesives. Chapter 15 reviews orthopaedic biomaterials, which is currently one of the most dominating areas of research in the biomaterial market. Interactions between various biomaterials such as ceramics and metals are explained in detail in this chapter. The rest of the chapters review tissue adhesives and cements utilized in surgical procedures along with bonding agents used in bone and tooth repair.

In summary, we believe “Joining and assembly of medical materials and devices” is the first major text that assimilates a wide range of biomaterial concepts with respect to medical device assembly. This book is well edited and very easy to read. The editors should be commended on their ability to simplify complex biomaterial concepts through the use of schematic diagrams and tables. Apart from engineers and researchers, this book should be in every biomedical or biomaterials professor’s library. This book can be used to develop an advanced graduate level course on biomaterials or medical device assembly on its own or in conjunction with Biomaterials Science: An Introduction to Materials in Medicine by Ratner et al., 2012 (ISBN-10: 0123746264) [2]. Due to the complexity of this subject and an exorbitant cost of $290, the book may be limited to a specific audience that has previous knowledge in this subject matter. As technology continues to develop, this book will require continued revisions as continued research will inevitably lead to newer advances in the world of medical devices. For the next edition, the authors should consider adding a chapter on assembling textile biomaterials and tissue stapling devices which are used increasingly in bariatric and vascular surgeries. But until this time comes, “Joining and assembly of medical materials and devices” is the new gold standard for understanding the concepts and assembly of medical materials and devices. We thank the biomedical engineering online editors for inviting us to write this review.

## Competing interests

Both authors declare no competing interest with regards to this invited review.

## Authors’ contributions

The authors contributed equally to the manuscript. Both authors read and approved the final manuscript.
